# Osteopontin as Candidate Biomarker of Coronary Disease despite Low Cardiovascular Risk: Insights from CAPIRE Study

**DOI:** 10.3390/cells11040669

**Published:** 2022-02-15

**Authors:** Federico Carbone, Jennifer Meessen, Marco Magnoni, Daniele Andreini, Aldo Pietro Maggioni, Roberto Latini, Fabrizio Montecucco

**Affiliations:** 1 First Clinic of Internal Medicine, Department of Internal Medicine, University of Genoa, 6 Viale Benedetto XV, 16132 Genoa, Italy; federico.carbone@unige.it; 2 IRCCS Ospedale Policlinico San Martino, Genoa—Italian Cardiovascular Network, 10 Largo Benzi, 16132 Genoa, Italy; 3 Department of Cardiovascular Research, IRCCS—Istituto di Ricerche Farmacologiche Mario Negri, 19 Via Giuseppe La Masa, 20156 Milan, Italy; jennifer.meessen@marionegri.it (J.M.); roberto.latini@marionegri.it (R.L.); 4 IRCCS Ospedale San Raffaele, 20132 Milan, Italy; magnoni.marco@hsr.it; 5 Centro Cardiologico Monzino, IRCCS, 20138 Milan, Italy; daniele.andreini@unimi.it; 6 Department of Biomedical and Clinical Sciences “Luigi Sacco”, University of Milan, 20157 Milan, Italy; 7 ANMCO Research Center, Heart Care Foundation, 50121 Florence, Italy; maggioni@anmco.it

**Keywords:** cardiovascular risk, osteopontin, coronary artery disease, inflammation, neutrophil

## Abstract

Stratification according high cardiovascular (CV) risk categories, still represents a clinical challenge. In this analysis of the CAPIRE study (NCT02157662), we investigate whether inflammation could fit between CV risk factors (RFs) and the presence of coronary artery disease (CAD). In total, 544 patients were included and categorized according with the presence of CAD and CV risk factor burden (low/multiple). The primary endpoint was to verify any independent association of neutrophil-related biomarkers with CAD across CV risk categories. The highest values of osteopontin (OPN) were detected in the low RF group and associated with CAD (23.2 vs. 19.4 ng/mL; *p* = 0.001), although no correlation with plaque extent and/or composition were observed. Conversely, myeloperoxidase (MPO) and resistin did not differ by CAD presence. Again, OPN was identified as independent variable associated with CAD but only in the low RF group (adjOR 8.42 [95% CI 8.42–46.83]; *p*-value = 0.015). As an ancillary finding, a correlation linked OPN with the neutrophil degranulation biomarker MPO (*r* = 0.085; *p* = 0.048) and resistin (*r* = 0.177; *p* = 3.4 × 10^−5^). In the present study, OPN further strengthens its role as biomarker of CAD, potentially bridging subclinical CV risk with development of atherosclerosis.

## 1. Introduction

Despite advances in diagnostic tools, stratifying patients according with cardiovascular (CV) risk still represents a clinical challenge. Since the paradigm shift from vulnerable plaque to the vulnerable patient took place in early 2000s [1], the need to standardize and then individualize CV risk factors (RFs) assessment has become a priority [2,3]. However, the concept of “individualized total risk estimation” is perhaps a misnomer [4]. No risk estimation score accommodates all the known RFs. Some RFs may still be unknown, and for those that are known, definitions and phenotyping are constantly evolving [5,6,7,8]. In this view, novel and more specific RFs are needed. The Coronary Atherosclerosis in Outlier Subjects: Protective and Novel Individual Risk Factors Evaluation (CAPIRE) study represents an intriguing model in which “outlier” patients with unexpected coronary artery disease(CAD) despite a low risk profile and those without CAD (or mild disease only) but multiple RFs are identified by coronary computed tomography angiography (CCTA) [9]. This approach would enable a preliminary investigation into where inflammatory molecules lie between CAD and CV RFs. Indeed, inflammation remains one of the strongest CV risk modifiers, but the sources—likely local and systemic—and the extent to which they contribute to CV risk burden are not yet clear [10]. Here, we focused on myeloperoxidase (MPO), resistin, and osteopontin (OPN), widely recognized as hallmark of neutrophil activation and chemotaxis. Especially, OPN is emerging as a promising candidate due to its pleiotropic properties and the effects on both metabolic and cardiovascular unbalance and innate immune response as well [11,12]. Historically known to modulate bone remodeling, OPN exerts a regulatory role on immune response in several classes of diseases. Therefore, in the present study, we aimed at preliminary investigating inflammatory biomarkers related to neutrophil activation—with a special focus on osteopontin (OPN), MPO and resistin—to verify their accuracy toward the identification of CAD in relation to or despite the presence of RFs.

## 2. Materials and Methods

### 2.1. Enrolled Subjects and Study Design

CAPIRE study (NCT02157662)—outlier project by the Gruppo Italiano per lo Studio della Sopravvivenza nella Insufficienza Cardiaca (GISSI)—enrolled 544 patients between January 2011 and June 2013, as previously described [9]. All those patients are finally included in the present analysis. CAPIRE study was specifically designed as a prospective observational multicenter study aiming at comparing extreme selected population according with CCTA and risk profile. CAD was defined according with segment involvement score 8SIS) at CCTA (>5 of the 16 segments defined by the American Heart Association classification) [13]. RFs burden was based on the Adult Treatment Panel III and 2013 American College of Cardiology/American Heart Association (AHA) guidelines for cardiovascular prevention [14,15]. It was then categorized as low (no or one RF with the exclusion of patients with type 1 or 2 diabetes as single RF) or multiple (≥2 RFs). Four groups were then identified: no-CAD/low RF, CAD/multiple RFs, and the outlier groups CAD/low RF and no-CAD/multiple RFs. Inclusion and exclusion criteria have been illustrated elsewhere [9].

Peripheral venous blood samples were drawn in all patients and immediately centrifuged. Separate aliquots of plasma were then stored at −70 °C in a dedicated biological bank. Biochemical variables were measured with standard automated laboratory methods. Colorimetric enzyme-linked immunosorbent assay (ELISA) was used to assay plasma levels of MPO, resistin and OPN (all from R&D systems, Minneapolis, MN, USA) in a central laboratory by personal unaware of patient’s characteristics. The lower detection limits were 62.50 pg/mL for MPO and OPN, and 31.25 pg/mL for resistin. Mean intra- and inter-assay coefficients of variation were <10% for both.

### 2.2. Endpoint Adjudication and Power Study Calculation

The primary endpoint of the study was to verify whether the abovementioned hallmarks of neutrophil activation/chemotaxis (i.e., MPO, resistin, and OPN) were independently association with the presence of CAD in low/multiple RF groups. Secondary endpoint was the search for any potential correlation between OPN and surrogate markers of neutrophil degranulation. Study power calculation was estimated on the primary outcome with an expected 2-fold higher risk of having CAD associated with OPN increase [16]. Our sample size in the low RF group (*n* = 322) was finally sufficient to reach a study power of 80% with a two-sided alpha error of 5%.

### 2.3. Statistical Analysis

Continuous variables are expressed as median and interquartile range [IQR] or mean ± standard deviation (SD) according to their distribution assessed with the Kolmogorov–Smirnov test. Categorical variables are, instead, presented as absolute and relative (%) frequencies. Intergroup comparisons were assessed by one-way ANOVA, Mann–Whitney or χ2 tests for continuous and categorical parameters, respectively. Linear relationships were instead determined by Spearman’s rank correlation test. Univariate and adjusted logistic regression models (presented as odds ratio [OR] and 95% confidence interval [CI]) were used to assess CAD. Being not normally distributed, OPN was log-transformed before including it in the regression analyses. Multivariate analysis was built by including age, sex, body mass index, systolic blood pressure and antiplatelet use, high-density lipoprotein cholesterol, triglycerides, and creatinine. Post hoc receiver operating characteristic (ROC) curve analysis was then used to further estimate the accuracy of OPN. The area under the curve (AUC) was presented with 95% CIs, whereas the cut-off point was calculated by maximizing the Youden index. The analyses were performed using SPSS version 27 (Armonk, NY, USA). For all the analyses, a two-sided *p* < 0.05 value was considered as statistically significant.

## 3. Results

### 3.1. Clinical, Biochemical Variables and Echocardiographic Assessment of the Study Cohort

In this cross-sectional analysis, 544 patients were enrolled. When categorized for RF burden, patients were equally distributed for age, sex, and serum levels of total and low-density lipoprotein cholesterol. Regarding inflammatory biomarkers, only MPO was significantly associated with the RF burden (median 141 vs. 99.3 ng/mL; *p* = 4 × 10^−6^) (Table S1).

Not surprisingly, age and male sex were associated with the presence of CAD in both low/multiple RF groups, whereas the association of CAD with BMI, hypertension, high density lipoprotein cholesterol (HDL-c), triglycerides and renal function reached statistical significance in the low RF group only (Table 1).

The highest values of OPN were detected in the low RF group and associated with CAD (median 23.2 vs. 19.4 ng/mL; *p* = 0.001) (Table 1). Even if not statistically significant, OPN was 9% higher in CAD vs no-CAD in the high RF group. No differences by CAD (yes/no) were found for MPO or resistin. Of interest, MPO was consistently higher in the multiple RF group than in the low RF group, indicating that RFs, rather than CAD, were driving MPO (Table 2). When outliers were compared, OPN revealed a prevalent association with the presence of CAD rather than the RF burden (23.2 vs. 18.8 ng/mL; *p* = 0.007). Nevertheless, when CAD extent and composition were considered—independently of RF burden—circulating levels of OPN did not differ across groups (Table S2).

### 3.2. OPN Is Only Partially Associated with Neutrophil Degranulation Biomarkers

When the overall cohort was considered, OPN showed a positive correlation with age, which was generally maintained across the study groups (Table 2).

About other neutrophil-related biomarkers, the correlation of OPN with MPO was weak (*r* = 0.085; *p* = 0.048) and not confirmed across the study groups. The association between OPN and resistin was instead stronger (*r* = 0.177; *p* = 3.4 × 10^−5^) and maintained across the study groups (except for the low-risk CAD group).

### 3.3. OPN Is Independently Associated with CAD in the Outlier Group of Low Risk Factor

When limited to the low RF group, high OPN levels were associated with about 10-fold odds of having CAD at logistic regression analysis (OR 9.22 [95% CI 2.39–35.54]; *p* = 0.001). This strong association was confirmed even after adjustment for confounding factors (i.e., age, sex, BMI, systolic BP, antiplatelet use HDL-c, triglycerides, and creatinine), thus maintaining an OR of 8.42 (95% CI 8.42–46.83); *p*-value = 0.015) (Table 3).

ROC curve analysis was then performed to test the accuracy of OPN at identifying the outlier group of patients with low RF burden but CAD. The AUC of ROC curve was 0.620 (95%CI: 0.547–0.686), whereas the best cut-off point identified was 22.05 ng/mL, being characterized by a specificity of 0.643 and a sensitivity of 0.615 (Figure S1).

## 4. Discussion

The main finding of the present study is the independent association between OPN and CAD in the subgroup of patients with low CV RFs. This very intriguing finding may be ascribed to the pleiotropic activity of OPN and needs to be discussed.

On the one hand, this association is not surprising as OPN is long-known to promote atherosclerotic plaque development and progression in several ways. Constitutively produced by M1 macrophages and up-regulated through IL-6 trans-signaling [17], OPN generates a positive feedback loop that sustains macrophage proliferation, polarization, activation, and chemotaxis in autocrine/paracrine manner [18,19,20]. We have previously demonstrated that high circulating levels of OPN are associated with features of plaque instability: increased lipid content, reduced smooth muscle cells, complement activation and pro-inflammatory infiltrate including M1-polarized macrophages, neutrophils, and related products (namely matrix metalloproteinase-9) [11,21]. These features add to the classic involvement of OPN in vascular calcification [22]. Nevertheless, we were unable to demonstrate any association between circulating OPN and CAD extent/severity. Also interesting is the effect of OPN on neutrophils chemotaxis [23,24,25]. This selective activity may explain the weak correlation with the neutrophil degranulation product MPO, whereas much stronger, was that with resistin, which would exert—although this is debated—an inhibitory effect on neutrophil chemotaxis [26,27,28]. Overall, both OPN and MPO lack of correlation with CAD presence/extent/severity. This negative finding may open intriguing scenarios on the role of neutrophil-related inflammation during the whole plaque lifespan [29,30,31,32].

On the other hand, it seems rather counterintuitive that the association between OPN and CAD is limited to the low RF group. Several lines of evidence indeed claim for OPN not only a role in CAD pathophysiology, but they rather propose it as biomarker and bridge of global CardioMetabolic risk [33]. OPN is up regulated within dysfunctional visceral adipose tissue, where it activates tissue macrophages and directly affects adipocyte function [34,35]. Ultimately, this would link OPN to chronic low-grade inflammation. In interventional studies, OPN would also serve as predictive marker of metabolic improvement in interventional studies [12,36], thus pointing out the need of considering qualitative rather than quantitative changes in body fat composition [5,6]. The study design of CAPIRE does not consider such emerging paradigm in CardioMetabolic RF definition and this may be considered the major limitation of the study, alongside with missing information about systemic inflammatory status (e.g., C-reactive protein). Furthermore, ROC curve analysis shows a low—although significant—accuracy of OPN at identifying the presence of CAD in the subgroup of patients with low RF burden.

However, interesting insights emerge from the low RF group. Whereas the study protocol excludes diabetes as single risk factor and does not consider insulin resistance, development of CAD shows an association with other metabolic syndrome determinants: higher BMI and triglycerides levels, and lower HDL-c and hypertension. This finding may lead to important considerations. First, the need for a paradigm shift from CV to CardioMetabolic risk [10,37]: this should drive future research questions and study designs. Therefore, in line with the hypothesis-generating aim of CAPIRE study, we would point out the need to fully consider CardioMetabolic risk according to new evidence: visceral adipose tissue distribution, ectopic fat deposition, low-grade inflammatory status, and insulin resistance as well. As second, the results of the present study suggest a detrimental role of neutrophil-related inflammation, even in outlier patients, in which current CV risk paradigm does not recognize a CAD risk [38]. Among the complex inflammatory network involving several classes of cytokines, here we provide indirect but confirmative data about the role of OPN as biomarker of CAD. Future studies are expected to confirm the intraplaque expression of OPN [39,40], also establishing whether associations exist with its circulating levels and plaque composition/vulnerability. Studies exploring the potential link between macrophages, OPN, and neutrophil activation are also expected.

In conclusion, the results of this hypothesis-generating study further confirm a role of OPN in CAD and provide stimulating challenges about the role of this pleiotropic inflammatory molecules in the context of CAD and global CV risk.

## Figures and Tables

**Table 1:** Characteristics of the study cohort stratified by the burden of risk factor (RFs) and the presence of coronary artery disease (CAD).

	Low RF			Multiple RF		
Parameter	CAD (SIS > 5) (*n* = 93)	No-CAD (*n* = 229)	*p*-Value	No-CAD (*n* = 120)	CAD (SIS > 5) (*n* = 102)	*p*-Value
Age, years	63.8 ± 7.2	57.5 ± 8.5	8.9 × 10^−10^	58.2 ± 8.0	62.5 ± 7.1	4.3 × 10^−5^
Sex, male	84 (90.3)	111 (48.5)	3.3 × 10^−12^	51 (42.5)	72 (70.6)	2.7 × 10^−5^
BMI, kg/m^2^	27.3 ± 4.2	25.2 ± 4.0	1.4 × 10^−5^	27.4 ± 3.9	28.2 ± 4.6	0.143
Family history of CAD, yes	9 (9.7)	23 (10.0)	0.921	78 (65.0)	59 (57.8)	0.274
Hypertension, yes	36 (38.7)	55 (24.0)	0.008	103 (85.8)	93 (91.2)	0.217
Current smoker, yes	8 (8.6)	15 (6.6)	0.517	55 (45.8)	55 (53.9)	0.230
Diabetes, yes	0	0	-	29 (24.2)	39 (38.2)	0.023
Systolic BP, mmHg	130.7 ± 16.0	125.0 ± 13.7	0.001	129.1 ± 14.6	135.2 ± 16.7	0.004
Antiplatelets, yes	31 (33.3)	25 (10.9)	2.0 × 10^−6^	30 (25.0)	59 (57.8)	6.5 × 10^−7^
Statins, yes	11 (11.8)	18 (7.9)	0.260	68 (56.7)	73 (71.6)	0.022
Total-c, mg/dL	203.3 ± 48.9	201.6 ± 38.7	0.839	217.7 ± 46.4	201.6 ± 48.9	0.076
LDL-c, mg/dL	131.6 ± 44.3	123.9 ± 36.8	0.326	137.0 ± 42.5	126.3 ± 47.2	0.222
HDL-c, mg/dL	47.7 ± 11.5	63.1 ± 21.2	8.7 × 10^−5^	53.6 ± 16.6	48.1 ± 12.5	0.068
Triglycerides, mg/dL	129.5 [90.5–227.5]	80.0 [62.0–115.0]	4.1 × 10^−5^	134.5 [94.0–208.3]	140.5 [99.3–205.5]	0.735
Serum creatinine, mg/dL	0.94 ± 0.12	0.83 ± 0.19	0.004	0.83 ± 0.20	0.90 ± 0.16	0.091
OPN, ng/dL	23.2 [16.8–29.7]	19.4 [14.3–25.3]	0.001	18.8 [14.7–25.7]	20.4 [14.4–29.7]	0.240
MPO, ng/dL	95.7 [48.5–193.2]	104.3 [60.2–185.5]	0.467	133.7 [78.2–307.0]	141.9 [76.4–279.5]	0.776
Resistin, ng/dL	14.2 [10.5–20.3]	13.1 [9.1–18.1]	0.212	13.8 [9.6–20.3]	14.3 [11.0–20.2]	0.383

Continuous data are presented as mean ±SD or median [interquartile range], according with their distribution. Categorical data are presented as absolute (relative) frequencies. *p*-value for one-way ANOVA, Mann–Whitney or χ^2^ tests, as appropriate. RF, risk factors; SIS, segment involvement score; BMI, body mass index; CAD, coronary artery disease; BP, blood pressure; total-c, total cholesterol; LDL-c, low-density lipoprotein cholesterol; HDL-c, high-density lipoprotein cholesterol; OPN, osteopontin; and MPO, myeloperoxidase.

**Table 2:** Correlation of osteopontin with clinical and laboratory variables.

Variables	Overall Cohort		High Risk No-CAD		Low Risk CAD		Low Risk No-CAD		High Risk CAD	
	*r*	*p*-value	*r*	*p*-value	*r*	*p*-value	*r*	*p*-value	*r*	*p*-value
Age	0.203	2.0 × 10^−6^	0.348	9.9 × 10^−5^	0.221	0.036	0.145	0.029	−0.045	0.655
Age of menopause	0.103	0.176	0.342	0.013	0.144	0.734	0.036	0.741	−0.001	0.998
BMI	−0.004	0.919	−0.059	0.523	−0.068	0.521	−0.006	0.933	−0.071	0.480
Waist circumference	0.011	0.803	−0.038	0.677	−0.090	0.394	0.064	0.337	−0.089	0.376
Systolic BP	−0.019	0.658	0.154	0.094	−0.206	0.051	−0.073	0.276	−0.078	0.435
Diastolic BP	−0.096	0.026	−0.047	0.613	−0.297	0.004	−0.034	0.608	−0.170	0.087
Creatinine	0.100	0.149	0.216	0.098	−0.177	0.359	−0.082	0.473	0.197	0.210
Total-c	−0.005	0.936	−0.049	0.696	0.151	0.394	0.018	0.860	−0.061	0.680
LDL-c	−0.011	0.874	−0.110	0.386	0.245	0.162	0.016	0.882	−0.074	0.639
HDL-c	−0.057	0.395	0.079	0.543	0.088	0.619	−0.195	0.076	−0.045	0.775
Triglycerides	−0.018	0.784	−0.225	0.079	−0.115	0.524	0.173	0.106	−0.163	0.280
MPO	0.085	0.048	0.141	0.125	0.080	0.451	0.064	0.340	0.113	0.257
Resistin	0.177	3.4 × 10^−5^	0.218	0.017	0.000	0.997	0.144	0.030	0.318	0.001

The *p*-values refer to the Spearman’s rank correlation test. OPN, osteopontin; BMI, body mass index; BP, blood pressure; total-c, total cholesterol; LDL-c, low-density lipoprotein cholesterol; HDL-c, high-density lipoprotein cholesterol; and MPO, myeloperoxidase.

**Table 3:** Logistic regression of osteopontin (OPN) and coronary artery disease (CAD) in patients with low number of risk factors (RFs).

	Univariate Analysis		Multivariate Analysis *	
Parameter	OR (95% CI)	*p*-Value	OR (95% CI)	*p*-Value
OPN	9.22 (2.39–35.54)	0.001	8.42 (1.51–46.83)	0.015

Since osteopontin (OPN) was not normally distributed, we log transformed OPN before including it in the regression analyses. * Adjusted for age, sex, body mass index, systolic blood pressure, anti-platelet use, high-density lipoprotein cholesterol, triglycerides, and creatinine.

## Data Availability

The data presented in this study are available on appropriate request from the corresponding author.

## Supplementary Materials

The following supporting information can be downloaded at: https://www.mdpi.com/article/10.3390/cells11040669/s1, Figure S1: ROC curve analysis testing the predictive role of osteopontin (OPN), toward the presence of coronary artery disease according with risk factor categorization; Table S1: Characteristics of the study cohort according with risk factor (RF) categorization; Table S2: Association between serum levels of osteopontin (OPN) and coronary atherosclerotic plaque extent/severity in coronary artery disease (CAD) groups (low/high risk factors).
