# Supplementary material for: Osteopontin as Candidate Biomarker of Coronary Disease despite Low Cardiovascular Risk: Insights from CAPIRE Study
Source: Cells. 2022 Feb 15;11(4):669. doi: 10.3390/cells11040669 (PMC8870389; doi:10.3390/cells11040669)
Supplement: Supplementary file 1 [file cells-11-00669-s001.zip › cells-1586225-supplementary.pdf]

# Supplementary Material

## Table S1. Characteristics of the study cohort according with risk factor (RF) categorization.

| Parameter | Low RF (n=322) | Multiple RF (n=222) | *p*-value |
|---|---|---|---|
| Age, years | 59.3 ± 8.6 | 60.2 ± 7.9 | 0.210 |
| Sex, male | 195 (60.6) | 123 (55.4) | 0.231 |
| BMI, kg/m$^2$ | 25.8 ± 4.0 | 27.8 ± 4.3 | $1.5x10^{-7}$ |
| Family history of CAD, yes | 32 (9.9) | 137 (61.7) | $1.2x10^{-37}$ |
| Hypertension, yes | 91 (28.3) | 196 (88.3) | $3.2x10^{-43}$ |
| Current smoker, yes | 23 (7.1) | 110 (49.5) | $1.2x10^{-29}$ |
| Diabetes, yes | 0 (0.0) | 68 (30.6) | $2.5x10^{-26}$ |
| Systolic BP, mmHg | 126.7 ± 14.6 | 131.9 ± 15.8 | $7.9x10^{-5}$ |
| Antiplatelets, yes | 56 (17.4) | 89 (40.1%) | $4.0x10^{-9}$ |
| Statins, yes | 29 (9.0) | 141 (63.5%) | $2.0x10^{-41}$ |
| Total-c, mg/dL | 202.1 ± 41.5 | 210.9 ± 47.9 | 0.127 |
| LDL-c, mg/dL | 126.4 ± 39.1 | 132.7 ± 44.5 | 0.237 |
| HDL-c, mg/dL | 58.6 ± 20.1 | 51.3 ± 15.2 | 0.003 |
| Triglycerides, mg/dL | 91 [65 – 135] | 139 [97 – 207] | $7.1x10^{-8}$ |
| Serum creatinine, mg/dL | 0.86 ± 0.18 | 0.86 ± 0.19 | 0.907 |
| OPN, ng/mL | 20.3 [14.8 – 27.0] | 19.6 [14.5 – 27.5] | 0.844 |
| MPO, ng/mL | 99.3 [56.6 – 186.3] | 140.8 [78.2 – 283.6] | $4.0x10^{-6}$ |
| Resistin, ng/mL | 13.3 [9.4 – 19.0] | 13.9 [10.3 – 20.3] | 0.101 |

Continuous data are presented as mean ±SD or median [interquartile range], according with their distribution. Categorical data are presented as absolute (relative) frequencies. P-value for one-way ANOVA, Mann-Whitney or χ2 tests, as appropriate. RF: risk factors; BMI: body mass index; CAD: coronary artery disease; BP: blood pressure; total-c: total cholesterol; LDL-c: low-density lipoprotein cholesterol; HDL-c: high-density lipoprotein cholesterol; OPN: osteopontin; MPO: myeloperoxidase.

## Table S2. Association between serum levels of osteopontin (OPN) and coronary atherosclerotic plaque extent/severity in coronary artery disease (CAD) groups (low/high risk factors).

| | OPN (ng/mL) | p-value |
|---|---|---|
| **CAD extent** | | |
| <7 segments affected (n=91) | 22.2 [16.6 – 29.7] | 0.859 |
| >7 segments affected (n=102) | 22.8 [14.6 – 29.8] | |
| | | |
| **CAD severity** | | |
| No segment >50% affected (n=182) | 22.6 [15.7 – 29.8] | 0.210 |
| Any segment >50% affected (n=10) | 18.1 [10.5 – 29.8] | |
| | | |
| **Plaque composition** | | |
| >50% non-calcified (n=11) | 23.4 [13.8 – 30.3] | 0.265 |
| >50% calcified (n=130) | 23.0 [16.7 – 30.6] | |
| Mixed (n=53) | 19.9 [13.9 – 27.8] | |

P-value for one-way ANOVA or Mann-Whitney tests, as appropriate.

**Figure S1. ROC curve analysis testing the predictive role of osteopontin (OPN), toward the presence of coronary artery disease according with risk factor categorization.**

| | Discriminatory power within Low RF, outcome = CAD | Discriminatory power within multiple RF, outcome = CAD | |
|---|---|---|---|
| **OPN** | 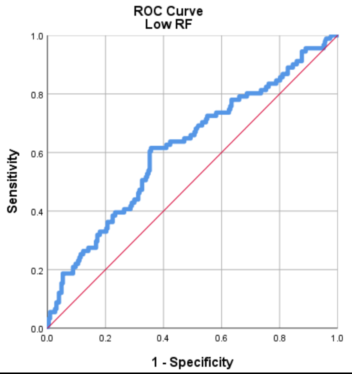  | 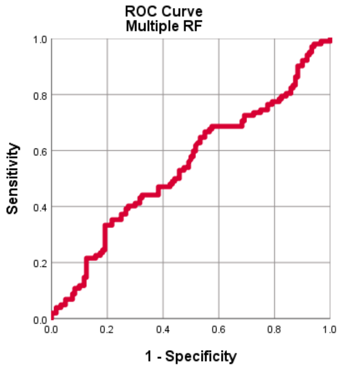  | 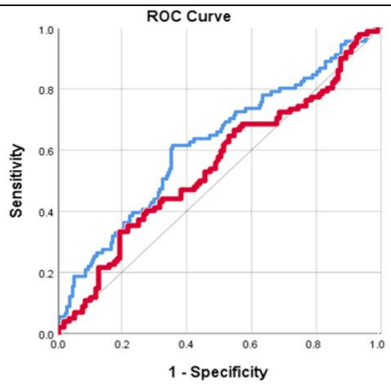  |
| | Area under the curve: 62%, Optimal cut-off: 22.05 | Area under the curve: 55%, Optimal cut-off: 26.84 | |

**Figure S1. ROC curve analyses.** ROC curve analyses testing the accuracy of osteopontin (OPN) toward the presence of coronary artery disease (CAD) according with risk factor (RF) categorization.

**Appendix**

Steering Committee

A. Maseri † (Chairman; Firenze), D. Andreini (Milano), S. Berti (Massa), M. Canestrari (Fano), G. Casolo (Lido di Camaiore), D. Gabrielli (Roma), R. Latini (Milano), M. Magnoni (Milano), P. Marraccini (Pisa), T. Moccetti (Lugano), M.G. Modena (Modena)

Coordinating Center: A.P. Maggioni, M. Gorini, F. Bianchini, I. Cangioli, A. Lorimer (Centro Studi ANMCO Firenze)

Imaging Core Laboratory: D. Andreini, G. Pontone, E. Conte (Centro Cardiologico Monzino Milano)

Centralized biobank and biomarker core laboratory: D. Novelli, F. Gaspari, S. Ferrari, A. Cannata, N. Stucchi, M. Fois, R. Bernasconi, G. Balconi (Istituto Mario Negri, Milano and Bergamo), T. Vago, T. Letizia (Ospedale Luigi Sacco, Milano), B. Bottazzi, R. Leone (Istituto Clinico Humanitas, Rozzano).

Central ECG Reading: I. Suliman (Centro Studi ANMCO, Firenze)

Psychologists CRF Group: M. Sommaruga † (IRCCS Salvatore Maugeri Unità di Psicologia, Milano), P. Gremigni (Dipartimento di Psicologia Università di Bologna)

Participating Centers and Investigators

Fano, Ospedale S Croce (R. Olivieri); Fermo, Ospedale Civile A. Murri (L. Pennacchietti); Lido di Camaiore, Nuovo Ospedale Versilia (M. Magnacca); Lugano, Cardiocentro Ticino (M.G. Rossi, E. Pasotti, T. Moccetti); Massa, FTGM - Stabilimento di Massa (A. Clemente); Milano, Centro Cardiologico Monzino (D. Andreini, G. Pontone, S. Mushtaq); Modena, Ospedale Policlinico (E. Mauro, G. Boriani); Parma, AOU. di Parma (F. Pigazzani); Pisa, AOU Pisana (L. Faggioni); Pisa, FTGM - Stabilimento di Pisa (M. Ciardetti); Udine, AOU SM della Misericordia (M. Puppato

**Centro di Coordinamento**
**Centro Studi ANMCO**

Via La Marmora, 34
50121 Firenze - Italia
Tel. +39 055 5101354
Fax +39 055 5101310
